# 2-Methyl-4-nitro­phenol

**DOI:** 10.1107/S1600536809018716

**Published:** 2009-05-23

**Authors:** Sheng Bi, Yong-Zhong Wu, Yi-Xin Zhou, Jian-Guo Tang, Cheng Guo

**Affiliations:** a College of Science, Nanjing University of Technology, Xinmofan Road No. 5 Nanjing, Nanjing 210009, People’s Republic of China; b Department of Applied Chemistry, Nanjing College of Chemical Technology, Geguan Road No. 625 Dachang District Nanjing, Nanjing 210048, People’s Republic of China

## Abstract

The mol­ecule of the title compound, C_7_H_7_NO_3_, is nearly planar [maximum deviation 0.112 (3) Å for one of the notro O atoms]. In the crystal structure, inter­molecular O—H⋯O and C—H⋯O inter­actions link the mol­ecules into a three-dimensional network.

## Related literature

For a related structure, see: Ahmed & Ashwini (2004 ▶). For bond-length data, see: Allen *et al.* (1987 ▶).
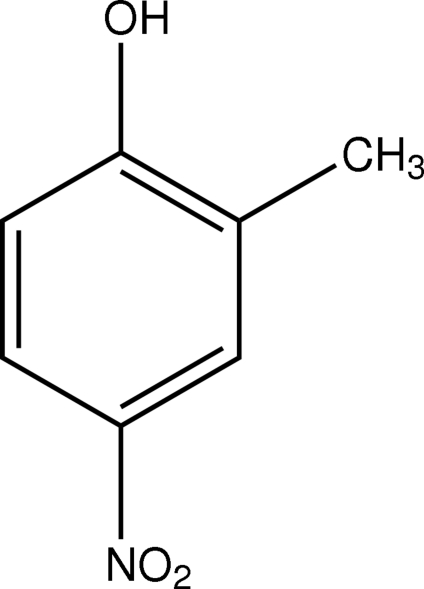


## Experimental

### 

#### Crystal data

C_7_H_7_NO_3_
*M* *_r_* = 153.14
Monoclinic, 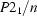

*a* = 5.6210 (11) Å
*b* = 8.7420 (17) Å>
*c* = 14.300 (3) Å
β = 100.71 (3)°
*V* = 690.4 (2) Å^3^
*Z* = 4
Mo *K*α radiation
μ = 0.12 mm^−1^
*T* = 294 K
0.30 × 0.20 × 0.10 mm

#### Data collection

Enraf–Nonius CAD-4 diffractometer
Absorption correction: ψ scan (North *et al.*, 1968 ▶) *T*_min_ = 0.966, *T*_max_ = 0.988
1378 measured reflections
1245 independent reflections
870 reflections with *I* > 2σ(*I*)
*R*_int_ = 0.027
3 standard reflections frequency: 120 min intensity decay: 1%

#### Refinement

*R*[*F* ^2^ > 2σ(*F* ^2^)] = 0.054
*wR*(*F* ^2^) = 0.181
*S* = 1.01
1245 reflections
102 parameters
H-atom parameters constrained
Δρ_max_ = 0.25 e Å^−3^
Δρ_min_ = −0.24 e Å^−3^

### 

Data collection: *CAD-4 Software* (Enraf–Nonius, 1989 ▶); cell refinement: *CAD-4 Software*; data reduction: *XCAD4* (Harms & Wocadlo, 1995 ▶); program(s) used to solve structure: *SHELXS97* (Sheldrick, 2008 ▶); program(s) used to refine structure: *SHELXL97* (Sheldrick, 2008 ▶); molecular graphics: *ORTEP-3 for Windows* (Farrugia, 1997 ▶); software used to prepare material for publication: *SHELXL97*.

## Supplementary Material

Crystal structure: contains datablocks global, I. DOI: 10.1107/S1600536809018716/hk2688sup1.cif

Structure factors: contains datablocks I. DOI: 10.1107/S1600536809018716/hk2688Isup2.hkl

Additional supplementary materials:  crystallographic information; 3D view; checkCIF report

## Figures and Tables

**Table 1:** Hydrogen-bond geometry (Å, °)

*D*—H⋯*A*	*D*—H	H⋯*A*	*D*⋯*A*	*D*—H⋯*A*
O3—H3*A*⋯O2^i^	0.82	2.10	2.770 (4)	138
C7—H7*C*⋯O1^ii^	0.96	2.57	3.505 (5)	165

Symmetry codes: (i) 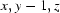; (ii) 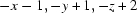.

**Fig. 1.**
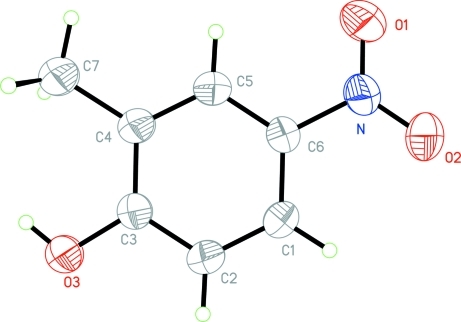
The molecular structure of the title molecule, with the atom-numbering scheme. Displacement ellipsoids are drawn at the 30% probability level.

**Fig. 2.**
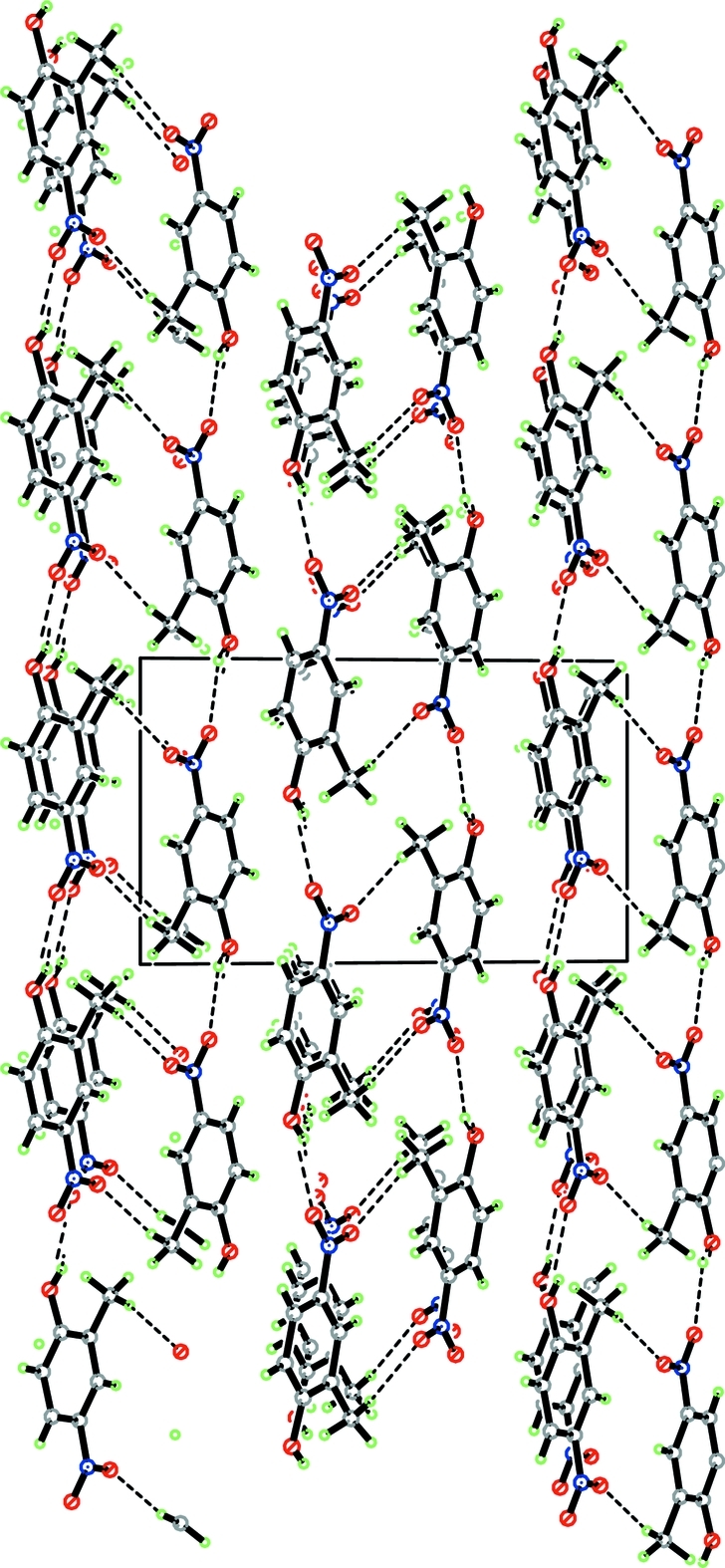
A partial packing diagram of the title compound. Hydrogen bonds are shown as dashed lines.

## supplementary crystallographic information

### Comment

Some derivatives of benzoic acids are important chemical materials. We report herein the crystal structure of the title compound.

In the molecule of the title compound (Fig 1), the bond lengths (Allen *et al.*,  1987) and angles are within normal ranges. Ring A (C1-C6) is, of course, planar. Atoms O1, O2, O3, N and C7 are 0.112 (3), 0.023 (3), 0.049 (3), 0.026 (4) and -0.042 (3) Å away from the ring plane, respectively. So, the molecule is nearly planar.

In the crystal structure, intermolecular O-H···O and C-H···O interactions (Table 1) link the molecules into a network, in which they may be effective in the stabilization of the structure.

### Experimental

For the preparation of the title compound, ethyl acetate (150 ml), 2-methyl-phenol (5.9 g) and zinc chloride (7.4 g) are placed in an ultrasonic cleaning bath equipped with a round botton flask, and then nitric acid (5.9 g) was added dropwise in 3 min. After the reaction was completed, water (200 ml) was added. After evaporation of the organic layer, the obtained product (Ahmed & Ashwini, 2004) was crystallized by slow evaporation of a methanol solution.

### Refinement

H atoms were positioned geometrically, with O-H = 0.82 Å (for OH) and C-H = 0.93 and 0.96 Å for aromatic and methyl H, respectively, and constrained to ride on their parent atoms, with U_iso_(H) = xU_eq_(C,O), where x = 1.2 for aromatic H and x = 1.5 for all other H atoms.

### Crystal data

**Table:**

C_7_H_7_NO_3_	*F*(000) = 320
*M**_r_* = 153.14	*D*_x_ = 1.473 Mg m^−^^3^
Monoclinic, *P*2_1_/*n*	Mo *K*α radiation, λ = 0.71073 Å
Hall symbol: -P 2yn	Cell parameters from 25 reflections
*a* = 5.6210 (11) Å	θ = 9–13°
*b* = 8.7420 (17) Å	µ = 0.12 mm^−^^1^
*c* = 14.300 (3) Å	*T* = 294 K
β = 100.71 (3)°	Block, colorless
*V* = 690.4 (2) Å^3^	0.30 × 0.20 × 0.10 mm
*Z* = 4	

### Data collection

**Table:**

Enraf–Nonius CAD-4 diffractometer	870 reflections with *I* > 2σ(*I*)
Radiation source: fine-focus sealed tube	*R*_int_ = 0.027
graphite	θ_max_ = 25.3°, θ_min_ = 2.7°
ω/2θ scans	*h* = 0→6
Absorption correction: ψ scan (North *et al.*, 1968)	*k* = 0→10
*T*_min_ = 0.966, *T*_max_ = 0.988	*l* = −17→16
1378 measured reflections	3 standard reflections every 120 min
1245 independent reflections	intensity decay: 1%

### Refinement

**Table:**

Refinement on *F*^2^	Secondary atom site location: difference Fourier map
Least-squares matrix: full	Hydrogen site location: inferred from neighbouring sites
*R*[*F*^2^ > 2σ(*F*^2^)] = 0.054	H-atom parameters constrained
*wR*(*F*^2^) = 0.181	*w* = 1/[σ^2^(*F*_o_^2^) + (0.08*P*)^2^ + 0.74*P*] where *P* = (*F*_o_^2^ + 2*F*_c_^2^)/3
*S* = 1.01	(Δ/σ)_max_ < 0.001
1245 reflections	Δρ_max_ = 0.25 e Å^−^^3^
102 parameters	Δρ_min_ = −0.24 e Å^−^^3^
0 restraints	Extinction correction: *SHELXL97* (Sheldrick, 2008), Fc^*^=kFc[1+0.001xFc^2^λ^3^/sin(2θ)]^-1/4^
Primary atom site location: structure-invariant direct methods	Extinction coefficient: 0.017 (4)

### Special details

**Table:**

Geometry. All e.s.d.'s (except the e.s.d. in the dihedral angle between two l.s. planes) are estimated using the full covariance matrix. The cell e.s.d.'s are taken into account individually in the estimation of e.s.d.'s in distances, angles and torsion angles; correlations between e.s.d.'s in cell parameters are only used when they are defined by crystal symmetry. An approximate (isotropic) treatment of cell e.s.d.'s is used for estimating e.s.d.'s involving l.s. planes.
Refinement. Refinement of *F*^2^ against ALL reflections. The weighted *R*-factor *wR* and goodness of fit *S* are based on *F*^2^, conventional *R*-factors *R* are based on *F*, with *F* set to zero for negative *F*^2^. The threshold expression of *F*^2^ > σ(*F*^2^) is used only for calculating *R*-factors(gt) *etc*. and is not relevant to the choice of reflections for refinement. *R*-factors based on *F*^2^ are statistically about twice as large as those based on *F*, and *R*- factors based on ALL data will be even larger.

### Fractional atomic coordinates and isotropic or equivalent isotropic displacement parameters (Å^2^)

**Table:**

	*x*	*y*	*z*	*U*_iso_*/*U*_eq_	
O1	−0.3440 (5)	0.6807 (3)	0.9304 (2)	0.0769 (9)	
O2	−0.0576 (5)	0.7542 (3)	0.8605 (2)	0.0682 (8)	
O3	0.0480 (4)	0.0536 (3)	0.81980 (17)	0.0567 (7)	
H3A	−0.0372	−0.0079	0.8417	0.085*	
N	−0.1770 (5)	0.6536 (3)	0.8896 (2)	0.0490 (8)	
C1	0.0652 (6)	0.4640 (4)	0.8248 (2)	0.0478 (9)	
H1A	0.1525	0.5429	0.8034	0.057*	
C2	0.1146 (6)	0.3143 (4)	0.8076 (2)	0.0487 (9)	
H2A	0.2352	0.2908	0.7734	0.058*	
C3	−0.0137 (6)	0.1979 (4)	0.8410 (2)	0.0417 (8)	
C4	−0.1941 (5)	0.2284 (3)	0.8932 (2)	0.0410 (8)	
C5	−0.2457 (6)	0.3796 (4)	0.9085 (2)	0.0415 (8)	
H5A	−0.3678	0.4038	0.9418	0.050*	
C6	−0.1175 (5)	0.4950 (4)	0.8747 (2)	0.0413 (8)	
C7	−0.3278 (6)	0.1019 (4)	0.9313 (3)	0.0531 (9)	
H7A	−0.2143	0.0360	0.9707	0.080*	
H7B	−0.4172	0.0441	0.8793	0.080*	
H7C	−0.4375	0.1443	0.9684	0.080*	

### Atomic displacement parameters (Å^2^)

**Table:**

	*U*^11^	*U*^22^	*U*^33^	*U*^12^	*U*^13^	*U*^23^
O1	0.093 (2)	0.0485 (16)	0.104 (2)	0.0137 (14)	0.0576 (18)	−0.0051 (15)
O2	0.0861 (19)	0.0303 (13)	0.097 (2)	−0.0030 (12)	0.0392 (16)	0.0063 (13)
O3	0.0661 (15)	0.0341 (13)	0.0802 (17)	0.0000 (11)	0.0405 (13)	−0.0053 (11)
N	0.0594 (18)	0.0337 (15)	0.0567 (17)	0.0039 (13)	0.0180 (14)	0.0005 (13)
C1	0.0539 (19)	0.0371 (17)	0.059 (2)	−0.0036 (14)	0.0281 (17)	0.0044 (15)
C2	0.0496 (19)	0.0413 (18)	0.063 (2)	−0.0036 (15)	0.0302 (17)	−0.0024 (16)
C3	0.0438 (17)	0.0339 (15)	0.0503 (18)	−0.0019 (14)	0.0168 (14)	−0.0029 (14)
C4	0.0389 (16)	0.0397 (17)	0.0473 (18)	−0.0032 (13)	0.0156 (14)	−0.0012 (14)
C5	0.0403 (16)	0.0407 (17)	0.0469 (17)	0.0021 (14)	0.0171 (14)	0.0005 (14)
C6	0.0461 (17)	0.0312 (16)	0.0500 (18)	0.0009 (13)	0.0177 (14)	−0.0006 (13)
C7	0.054 (2)	0.045 (2)	0.068 (2)	−0.0034 (15)	0.0285 (17)	0.0007 (16)

### Geometric parameters (Å, °)

**Table:**

O3—C3	1.357 (4)	C2—H2A	0.9300
O3—H3A	0.8200	C3—C4	1.393 (4)
N—O1	1.217 (3)	C4—C5	1.379 (4)
N—O2	1.225 (4)	C4—C7	1.496 (4)
N—C6	1.451 (4)	C5—C6	1.379 (4)
C1—C2	1.370 (5)	C5—H5A	0.9300
C1—C6	1.382 (4)	C7—H7A	0.9600
C1—H1A	0.9300	C7—H7B	0.9600
C2—C3	1.382 (4)	C7—H7C	0.9600
			
C3—O3—H3A	109.5	C5—C4—C7	121.1 (3)
O1—N—O2	122.9 (3)	C3—C4—C7	121.2 (3)
O1—N—C6	118.4 (3)	C6—C5—C4	120.5 (3)
O2—N—C6	118.7 (3)	C6—C5—H5A	119.8
C2—C1—C6	118.4 (3)	C4—C5—H5A	119.8
C2—C1—H1A	120.8	C5—C6—C1	121.6 (3)
C6—C1—H1A	120.8	C5—C6—N	119.9 (3)
C1—C2—C3	120.4 (3)	C1—C6—N	118.5 (3)
C1—C2—H2A	119.8	C4—C7—H7A	109.5
C3—C2—H2A	119.8	C4—C7—H7B	109.5
O3—C3—C2	115.9 (3)	H7A—C7—H7B	109.5
O3—C3—C4	122.7 (3)	C4—C7—H7C	109.5
C2—C3—C4	121.5 (3)	H7A—C7—H7C	109.5
C5—C4—C3	117.7 (3)	H7B—C7—H7C	109.5
			
O1—N—C6—C5	−1.5 (5)	O3—C3—C4—C5	−178.4 (3)
O2—N—C6—C5	178.6 (3)	C2—C3—C4—C5	1.8 (5)
O1—N—C6—C1	177.3 (3)	O3—C3—C4—C7	1.4 (5)
O2—N—C6—C1	−2.6 (5)	C2—C3—C4—C7	−178.3 (3)
C6—C1—C2—C3	−1.0 (5)	C3—C4—C5—C6	−1.5 (5)
C2—C1—C6—C5	1.2 (5)	C7—C4—C5—C6	178.6 (3)
C2—C1—C6—N	−177.5 (3)	C4—C5—C6—C1	0.0 (5)
C1—C2—C3—O3	179.7 (3)	C4—C5—C6—N	178.8 (3)
C1—C2—C3—C4	−0.5 (5)		

### Hydrogen-bond geometry (Å, °)

**Table:**

*D*—H···*A*	*D*—H	H···*A*	*D*···*A*	*D*—H···*A*
O3—H3A···O2^i^	0.82	2.10	2.770 (4)	138
C7—H7C···O1^ii^	0.96	2.57	3.505 (5)	165

Symmetry codes: (i) *x*, *y*−1, *z*; (ii) −*x*−1, −*y*+1, −*z*+2.
